# Non-invasive imaging of interstitial fluid transport parameters in solid tumors in vivo

**DOI:** 10.1038/s41598-023-33651-9

**Published:** 2023-05-02

**Authors:** Sharmin Majumder, Md Tauhidul Islam, Raffaella Righetti

**Affiliations:** 1 Department of Electrical and Computer Engineering, Texas A&M University, College Station, TX 77843 USA; 2 Department of Radiation Oncology, Stanford University, Stanford, CA 94305 USA

**Keywords:** Cancer imaging, Cancer microenvironment, Tumour biomarkers, Tumour heterogeneity, Diagnostic markers, Predictive markers, Prognostic markers, Biomedical engineering, Cancer, Biomarkers, Engineering

## Abstract

In this paper, new and non-invasive imaging methods to assess interstitial fluid transport parameters in tumors in vivo are developed, analyzed and experimentally validated. These parameters include extracellular volume fraction (EVF), interstitial fluid volume fraction (IFVF) and interstitial hydraulic conductivity (IHC), and they are known to have a critical role in cancer progression and drug delivery effectiveness. EVF is defined as the volume of extracellular matrix per unit volume of the tumor, while IFVF refers to the volume of interstitial fluid per unit bulk volume of the tumor. There are currently no established imaging methods to assess interstitial fluid transport parameters in cancers in vivo. We develop and test new theoretical models and imaging techniques to assess fluid transport parameters in cancers using non-invasive ultrasound methods. EVF is estimated via the composite/mixture theory with the tumor being modeled as a biphasic (cellular phase and extracellular phase) composite material. IFVF is estimated by modeling the tumor as a biphasic poroelastic material with fully saturated solid phase. Finally, IHC is estimated from IFVF using the well-known Kozeny–Carman method inspired by soil mechanics theory. The proposed methods are tested using both controlled experiments and in vivo experiments on cancers. The controlled experiments were performed on tissue mimic polyacrylamide samples and validated using scanning electron microscopy (SEM). In vivo applicability of the proposed methods was demonstrated using a breast cancer model implanted in mice. Based on the controlled experimental validation, the proposed methods can estimate interstitial fluid transport parameters with an error below 10% with respect to benchmark SEM data. In vivo results demonstrate that EVF, IFVF and IHC increase in untreated tumors whereas these parameters are observed to decrease over time in treated tumors. The proposed non-invasive imaging methods may provide new and cost-effective diagnostic and prognostic tools to assess clinically relevant fluid transport parameters in cancers in vivo.

## Introduction

**Figure 1:**
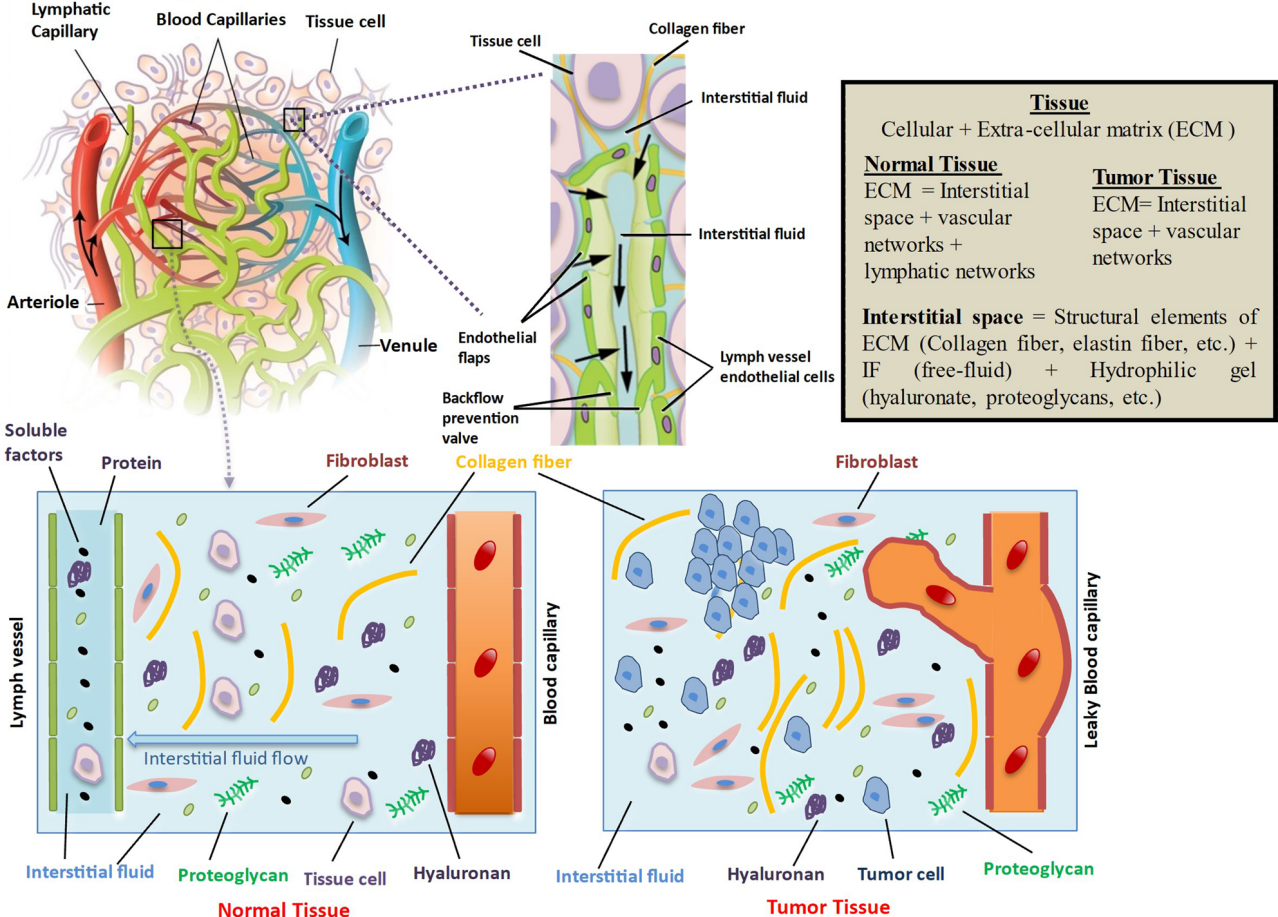
Pictorial representation of cellular, interstitial, vascular, and lymphatic networks in normal tissue (left) and tumors (right).

On a large scale (~ 1 mm), tissue is composed of interstitial matrix, cells, and the microvascular and lymphatic networks. The anatomical well-defined functioning lymphatic vessels present in normal tissues may be absent in solid tumors. Therefore, a solid tumor can be divided into three main sub-compartments: vascular, cellular, and interstitial^1^. The interstitial space, or interstitium, is a general term pertaining to the connective and supporting tissues of the body that are localized outside the blood and lymphatic vessels and parenchymal cells^2^. The interstitial space of tumors is composed predominantly of a collagen and elastic fiber network, which is the structural molecules of the interstitium or the extracellular matrix (ECM). Interspersed within this cross-linked structure are the interstitial fluid (IF) and the macromolecular constituents (hyaluronate and proteoglycans), which form a hydrophilic gel. IF is composed primarily of water and therefore is often assumed to be incompressible^1^. A graphical representation of normal tissue and tumor tissue consisting of cells, interstitial fluid, interstitium, vascular, and lymphatic networks on a macro scale is shown in Fig. 1.

Vascular and interstitial transport phenomena are clinically very relevant for progression and treatments. Once a molecule for cancer detection or treatment is injected into the blood stream, it encounters the following resistances before reaching the intracellular space: (a) transport through interstitial space and/or across cell membrane; (b) transport across microvascular wall^3^. Each of these transport processes may involve both convection and diffusion. Convection is proportional to the interstitial fluid velocity, which, in turn, is proportional to the pressure gradient in the interstitium. The proportionality constant, which relates fluid velocity to the pressure gradient, is referred to as the “interstitial hydraulic conductivity” (IHC), sometimes also referred to as “interstitial permeability”^4^, and it is measured in units of [m^2^/Pa.s]. Several factors such as elevated interstitial fluid pressure (IFP) and large transport distances in the interstitium can affect the efficacy of drug delivery to treat cancers^5^. IFP distribution is affected primarily by the characteristics of the tumor interstitium^6^. Khosravani et al.^7^ showed that the time course of IFP tracings, which may be substantially different from one tumor to the next, is strongly influenced by IHC. ECM may also contribute to the drug resistance of a solid tumor by preventing the penetration of therapeutic agents^6,8^. Therefore, investigation of interstitial mechanisms in cancers is important to understand and may improve drug delivery in solid tumors^5^.

In general, the tumor interstitial compartment is characterized by large interstitial space and large IHC compared to the host normal tissues^3,9–12^. In^3^, mean interstitial spaces for different types of tumors such as Fibrosarcoma 4956: 52.6%, W256 carcinoma: 36.3%, H5123 carcinoma: 43.3%, H3683 carcinoma: 50.6%, Novikoff hepatoma: 54.6%, Fibrosarcoma A-MC: 60%, Fibrosarcoma C-MC: 55%, etc. in rat host have been reported. Large interstitial space is, generally, due to the leakiness of lymphatic networks in tumor. In several tumors, collagen content of tumors was found to be higher than that of the host normal tissue^5^. On the other hand, hyaluronate and proteoglycans are usually present in lower concentrations in tumors. The large interstitial space and low concentrations of polysaccharides suggest that the values of IHC should be relatively high in tumors^5^. IHC range reported for colon adenocarcinoma LS174T tumors in vivo is (0.577–4.05) × 10^–13^ m^2^ (Pa.s)^−1^^13^. In^7^, IHC in vivo was reported as 3.22 × 10^–12^ m^2^ (Pa.s)^−1^ for clinically diagnosed cancer of the cervix, randomly selected from the Human cervix cancer.

Interstitial volume fraction (IVF) is usually obtained by subtracting the vascular volume fraction (VVF) (i.e., vascular space) from the extracellular volume fraction (EVF) (i.e., extracellular space). Typically, VVF is measured by a marker confined to blood vessels, and EVF is measured by a marker excluded by cells^3^. Several invasive methods have been considered to estimate IVF^11,12,14^. This include methods based on electron microscopy^12^ and methods based on injection of extracellular markers (such as sodium, chlorine, or d-mannitol) and vascular markers (such as dextran)^3,11,14^. Nuclear magnetic resonance (NMR) methods have been used to assess IVFs using intravenous injection of several agents^15–18^. In^19^, the authors established a method that allows two- and three-dimensional mapping of the tumor VVF and IVF during the same imaging session based on sequential injections of a large molecular weight (MW) and a low MW marker. However, several of these techniques are either not directly applicable to humans because of the unavailability of suitable vascular tracers or require extensive computational manipulation and ultrafast imaging to extract first pass kinetic principles^18^.

IHC has been estimated mostly under in vitro conditions and in vivo in a few studies^13,20^. In vitro, IHC is generally estimated by measuring fluid flow after applying pressure across a tissue slice of known area and thickness. In this case, hydration, slicing of the tissue and compression are potential factors that can influence the accuracy of IHC estimation^13^. In vivo estimation of IHC is not straightforward, but it can be obtained from the measurement of fluid velocity resulting from a natural or an applied pressure gradient^20–22^. In^8^, IHC was estimated from the transient stress relaxation rate by using a poro-viscoelastic model. Fluid reabsorption by blood vessels or lymphatics may result in an overestimation of IHC in vivo^1,21,23^. Fluid infusion techniques were used to assess IHC in several studies^6,13,24^. These techniques are dependent on the spherically symmetric distribution of the fluid infused and the heterogeneity in fluid flow. In^24^, IHC was estimated from dynamic contrast-enhanced (DCE)-MRI data set, which records the tissue uptake of a systemically delivered MRI-visible tracer (in this case Gd-DTPA). M. Milosevic et al.^6^ estimated IHC by fitting a spatio-temporal fluid dynamic model to the time course of IFP measurements. This model assumes that sudden insertion of the needle transiently perturbs the steady-state fluid balance, which recovers over time as a function of the vascular and interstitial hydraulic conductivities, the interstitial bulk modulus, and the extracellular, extravascular volume fraction. While several experiments of this sort are feasible in the laboratory, they are difficult to implement clinically in a manner that yields reliable results.

In the context of biomechanics, to evaluate tissue interstitial phenomena, two continuum theories are commonly employed: poroelasticity theory and/or mixture theory. The former assumes soft tissue to be a fluid-saturated poroelastic solid (a spongelike material) containing a uniform distribution of fluid source/sink points representing the transvascular flow to or from the interstitial compartment^1,25–28^. The latter models soft tissue as an intimate mixture of cellular phase and extracellular phases (a concentrated macromolecular solution)^29^. A porous medium is defined as a material volume consisting of solid matrix with interconnected voids^30^. Its porosity is the ratio of the void space to the total volume of the medium, and its hydraulic conductivity provides a measure of the flow conductivity in the porous medium. When tissue is compressed, fluid percolates through the pores of the sponge and translocate driven by a pressure gradient, which is generated by the deformation of the sponge. The resulting process is governed by the Darcy’s law, which depends on the pressure gradient, porosity, IHC, and interstitial fluid velocity^1^. Inspired by these theories, this paper proposes a non-invasive method to compute EVF, interstitial fluid volume fraction (IFVF), and IHC using ultrasound poroelastography (USPE). USPE is a non-invasive imaging modality that can be used to assess mechanical and transport properties of soft tissues by estimating the time-dependent strains generated in response to a small sustained compression^26,31^. EVF includes the volume of extracellular space (interstitial and vascular space), whereas IFVF includes the volume of interstitial fluid only^32,33^. To assess EVF, a solid tumor model is developed assuming a bi-phasic (cellular and extracellular phase) composite model. To assess IFVF, a bi-phasic poroelastic model with solid phase saturated by interstitial fluid is assumed. The well known Kozeny–Carman relation^34^ is used to assess IHC. Kozeny–Carman theory computes intrinsic permeability (*k,* related to IHC by viscosity of fluid) based on IFVF (defined as porosity in poroelastic theory), and cell diameter.

This study proposes novel methods to expand applications of elasticity imaging methods and identify new imaging markers of interstitial fluid transport mechanisms. Currently, there are no established non-invasive methods to directly image EVF, IVF, and IHC in tumors. These parameters are important to assess tumor aggressiveness and improve drug delivery to tumors. The proposed ultrasound-based methods are non-invasive, cost-effective and have the potential to be used in the clinics.

## Materials and methods

**Figure 2:**
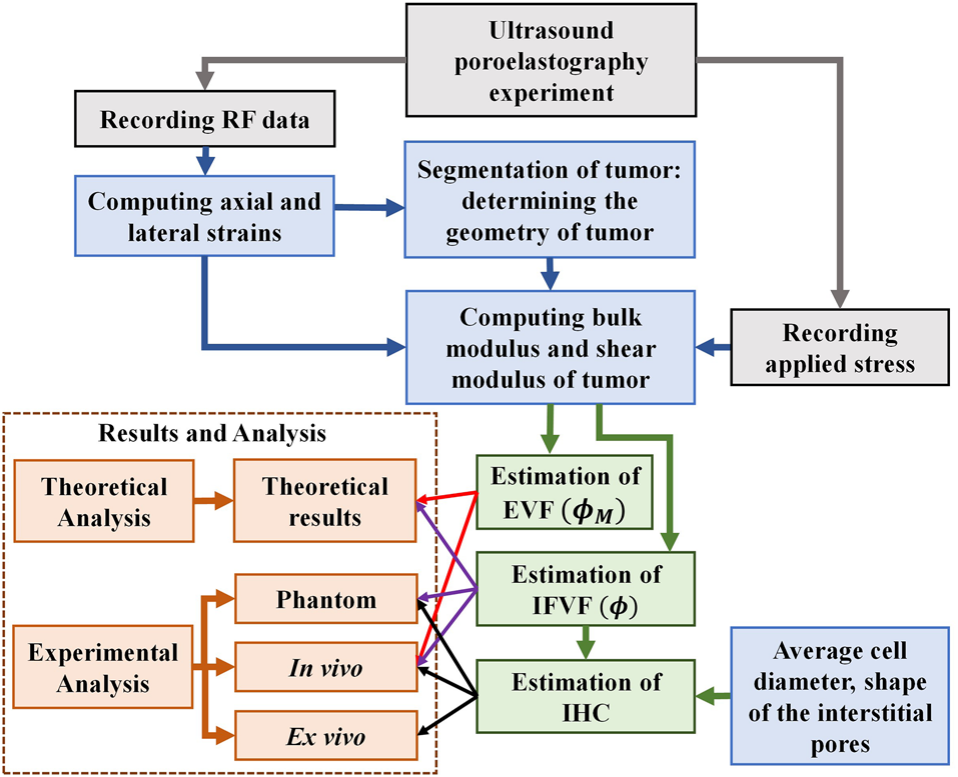
Workflow diagram of the proposed approach.

A step-by-step workflow of the proposed approach is shown in Fig. 2. Each step of the workflow is discussed in the next few paragraphs.

### Estimation of ${\varvec{I}}{\varvec{F}}{\varvec{V}}{\varvec{F}}({\varvec{\phi}})$

The solid tumor model developed herein is a bi-phasic (solid and fluid phase) poroelastic model that contains a uniform distribution of fluid source and/or sink channels. When an external compression is applied, a transient redistribution of the solid and fluid phases occurs. The interstitial fluid can either exude from the tumor surface or be reabsorbed into the capillary network. We will consider the ensemble of interstitial, vascular and cellular spaces as a continuous, deformable solid phase saturated with a fluid phase consisting of free to flow interstitial fluid (assumed to be incompressible). Solid phase is also assumed to be incompressible whereas the overall solid matrix is compressible^1,26^.

Our model for IFVF is built based on the theory developed for a fluid-saturated cracked solid by Budiansky and O’Connell^35^ for spherical pores. Based on the theory proposed in^35^, the relationship between the effective Poisson’s ratio (PR) of the material ($\nu$), effective shear modulus ($\mu$), shear modulus of the solid phase (${\mu }_{s}$), and cracked density ($\epsilon$) is defined as^35,36^,

1 $$\frac{\mu }{{\mu }_{s}}=\left[1-\frac{32}{45}\frac{\left(1-\nu \right)(5-\nu )}{(2-\nu )}\epsilon \right]$$

Cracked density ($\epsilon$), which is referred to the pore density in this study is defined as, $\epsilon =\frac{2N}{\uppi {V}_{b}}(\frac{{A}^{2}}{P})$. A is the area and P is the perimeter of the pores. $N/{V}_{b}$ is the number of pores in the bulk sample volume ${V}_{b}$. For circular cracks, $\epsilon$ reduces to

2 $$\epsilon =\left[\frac{45}{16}\frac{\left({\nu }_{s}-\nu \right)(2-\nu )}{\left(1-{\nu }^{2}\right)[10{\nu }_{s}-\nu \left(1+3{\nu }_{s}\right)]}\right]$$

where ${\nu }_{s} \mathrm{is}$ the PR of the solid phase. If the solid phase is assumed to be incompressible, $\frac{K}{{K}_{S}}$ tends to zero, which implies that ${\nu }_{s}\to 0.5$. Using this assumption and the elastic, isotropic equation $\nu =\frac{3K-2\mu }{6K+2\mu }$ into Eqs. (1) and (2), we get,

3 $$\frac{\mu }{{\mu }_{s}}=\frac{\left(\frac{3K-2\mu }{6K+2\mu }-5\right)\left(\frac{45}{16} \frac{3K-2\mu }{6K+2\mu }-\frac{45}{32}\right)\left(\frac{32}{45} \frac{3K-2\mu }{6K+2\mu }-\frac{32}{45}\right)}{\left({\left(\frac{3K-2\mu }{6K+2\mu }\right)}^{2}-1\right)\left(\frac{5}{2} \frac{3K-2\mu }{6K+2\mu }-5\right)}+1$$

where $K$ is the effective bulk modulus of the porous material, and ${K}_{S}$ is the bulk modulus of the solid phase. Mackenzie^37^ showed the relation between porosity ($\phi$, defined as IFVF in this paper), *K*, ${K}_{S}$, and ${\mu }_{s}$ of a homogeneous and isotropic elastic material containing spherical pores using

4 $$\frac{1}{K}=\frac{1}{(1-\phi ){K}_{S}}+\frac{3\phi }{4\left(1-\phi \right){\mu }_{s}}$$

Assuming $\frac{K}{{K}_{S}}\to 0$ simplifies Eq. (4) to

5 $${\mu }_{s}=\frac{3K\phi }{4(1-\phi )}$$

By solving (3) and (5), we obtain,

6 $$\mathrm{IFVF}, \phi =\left[\frac{40{\mu }^{2}+60K\mu }{45{K}^{2}+54K\mu +24{\mu }^{2}}\right]$$

### Estimation of ${{\varvec{E}}{\varvec{V}}{\varvec{F}}({\varvec{\phi}}}_{{\varvec{M}}})$

To compute EVF, we model the tumor as a biphasic (cellular phase and extracellular phase), isotropic, and elastic composite material^29^. Here, the composite theory developed by Weng et al.^38^ for multiphase composite material is used. Each phase is assumed to be uniform throughout the region of interest. Cellular and extracellular spaces are regarded as linear, isotropic elastic media, each characterized by two elastic parameters, Young’s modulus (YM) and PR (E, v) or, equivalently, the Lame’s parameters ($\lambda$, $\mu$). The key assumption is that the tissue consists of an array of incompressible cells with very low resistance to shear deformation^29^, surrounded by ECM consisting of an isotropic mesh of randomly oriented interconnected fibers. Tissue shear rigidity is assumed to result mainly from the extracellular matrix, which is treated as a compressible elastic mesh of interconnected fibers. The elastic properties of such a system were analyzed by Cox^39^ who showed that, the Lame’s parameters are equal for this material, ${\lambda }_{M}$ = ${\mu }_{M}$. This assumption was also made in^29^ as applied to tissues. The fact ${\lambda }_{M}$ = ${\mu }_{M}$ implies that the PR of the ECM is ${\nu }_{M}$ = ¼ where the subscript “M” stands for matrix (extracellular). Using ${\nu }_{M}$ = ¼ we can write, ${K}_{M}=\frac{5}{3}{\mu }_{M}$ where ${K}_{M}$ is the bulk modulus of the ECM material.

In^38^, for a 2-phase composite, the bulk and shear moduli reduce to the following equations (ECM is assumed as phase “0” and cellular phase is assumed as phase “1” denoted in the original model):

7 $$\frac{K}{{K}_{M}}=1+\frac{1- {\phi }_{\mathrm{M}}}{\frac{2\phi {K}_{M}}{3{K}_{M}+4{\mu }_{M}}+\frac{{K}_{M}}{{K}_{C}-{K}_{M}}}$$

8 $$\frac{\mu }{{\mu }_{M}}=1+\frac{1- {\phi }_{\mathrm{M}}}{\frac{6}{5}\frac{\phi ({K}_{M}+2{\mu }_{M})}{3{K}_{M}+4{\mu }_{M}}+\frac{{\mu }_{M}}{{\mu }_{C}-{\mu }_{M}}}$$

where *K*, $\mu$, ${\phi }_{\mathrm{M}}$ are the bulk modulus, shear modulus, and volume fraction of ECM, respectively. The subscript “*C*” stands for cellular phase. Using the cellular phase incompressibility assumption, ${K}_{c}$*→∞*, Eq. (7) reduces to

9 $$\frac{K}{{K}_{M}}=1+\frac{1- {\phi }_{\mathrm{M}}}{\frac{2\phi {K}_{M}}{3{K}_{M}+4{\mu }_{M}}}$$

Shear modulus of ECM is typically much higher than the shear modulus of cell in soft tissue^29^. Therefore, assuming ${\mu }_{c}\approx {0.1 \mu }_{M}$^29^, Eq. (8) reduces to,

10 $$\frac{\mu }{{\mu }_{M}}=1+\frac{1-{\phi }_{\mathrm{M}}}{\frac{6}{5}\frac{\phi ({K}_{M}+2{\mu }_{M})}{3{K}_{M}+4{\mu }_{M}}-\frac{10}{9}}$$

By solving Eqs. (9), and (10) using ${K}_{M}=\frac{5}{3}{\mu }_{M}$,

11 $${\phi }_{\mathrm{M}}=\left[\frac{ \left(-\left(15K+722\mu \right)\pm {\left(225{K}^{2}+207960K\mu +16384{\mu }^{2}\right)}^\frac{1}{2} \right) }{2(69K-187\mu )}\right]$$

Equation (11) has two solutions of ${\phi }_{\mathrm{M}}$. For the convenience of the analysis, we can parameterize Eq. (11) as, ${\phi }_{\mathrm{M}}=\left[\frac{ \left(-a\pm b \right) }{c}\right]$, where $a=\left(15K+722\mu \right)\ge 0$ and ${b=\left(225{K}^{2}+207960K\mu +16384{\mu }^{2}\right)}^\frac{1}{2}\ge 0$. The bound of ${\phi }_{\mathrm{M}}$ is $0\le {\phi }_{\mathrm{M}}\le 1$, which is $0\le \frac{ -a}{c}\pm \frac{ b }{c}\le 1$. Since $a\ge 0$ and $b\ge 0,$ if $c\ge 0$, the only option for ${\phi }_{\mathrm{M}}\ge 0$ is, ${\phi }_{\mathrm{M}}=\left[\frac{ \left(-a+ b \right) }{c}\right]$. Now, $c\ge 0$ if $2\left(69K-187\mu \right)\ge 0$, implies that $\mu \le \frac{ 69}{187}K$. If $\mu >\frac{ 69}{187}K$, then $c<0$. Upper limit of $\mu$ can be $1.5K$ because PR $(\nu )\ge 0$. For $\frac{69}{187}K<\mu <1.5K$, $c<0$, and $\frac{-a}{c}>1$. Therefore, the only way for ${\phi }_{\mathrm{M}}\le 1$ is ${\phi }_{\mathrm{M}}=\left[\frac{ \left(-a+ b \right) }{c}\right] .$ Therefore, we can ignore one solution and write ${\phi }_{\mathrm{M}}$ as,

12 $${\phi }_{\mathrm{M}}=\left[\frac{ \left(-\left(15K+722\mu \right)+ {\left(225{K}^{2}+207960K\mu +16384{\mu }^{2}\right)}^\frac{1}{2} \right) }{2(69K-187\mu )}\right]$$

### Estimation of interstitial hydraulic conductivity $({\varvec{I}}{\varvec{H}}{\varvec{C}})$

Numerous formulae that relate intrinsic permeability (*k*) of a porous media to various geometric properties of the pore space, such as porosity, pore-size distribution, specific surface, aspect ratio of pores, and tortuosity of passages, have been developed^40,41^. The Carman–Kozeny^42^ equation offers a useful approach to determine interstitial conductivity of tissue as studied by Levick as^40^,

13 $$k=\frac{{\phi }^{3}}{{c}_{kc}{(1-\phi )}^{2}{S}^{2}}$$

where $\phi$ is the porosity (defined as IFVF here) of the porous material, *S* is the wetted surface area per unit volume, and ${c}_{kc}$ is a dimensionless proportionality term, the Kozeny–Carman constant. The specific surface area per unit volume of solid grains is *S* = *2/r* assuming solid grain as ellipsoidal shape, where* r* is the average radius of the solid grain (radius of the cells, in our study). Average cell diameter was assumed to be 10 µm in^29^. In general, Kozeny factor (${c}_{kc}$) depends on channel shape and tortuosity. For the simplest case, straight cylindrical pores, ${c}_{kc}$ = 2, and for random porous beds where the void volume is less than 0 9, ${c}_{kc}$ is between 3 and 5^40^. In this study, we used ${c}_{kc}$ = 3 assuming random cylindrical pores in the tumor. IHC is then computed as *k* divided by IF viscosity ratio. Typically, the IF viscosity is assumed to be equal to the viscosity of water (850 Pa.s at 25 °C^43^) as IF consists mainly of water.

### Estimation of axial and lateral strain using USPE

Axial and lateral strains were computed from RF data using a previously developed DPHS and Kalman filtering based method^44^.

### Estimation of bulk modulus (${\varvec{K}}$) and shear modulus (${\varvec{\mu}}$) using USPE

Effective bulk modulus ($K$) and effective shear modulus ($\mu$) of the tumor are computed by knowledge of the YM and PR using the isotropic elastic constants conversion formula, $K=\frac{E}{6(0.5-\nu )}$ $,\mathrm{ and }\mu =\frac{E}{2(1+\nu )}$. To determine the YM and PR of the tumor and background tissue, we used our Eshelby-based method in^45^. In case of the in vivo experiments, the tumor shape Was approximated with its best fit ellipse using eigen decomposition^46–52^. Tumor boundaries were segmented from the axial strain elastograms.

### Computation of IFVF, EVF, IHC in cancer using USPE

Equations (6) and (12) are used to compute IFVF, and EVF, respectively, using $K$ and $\mu$ estimated by USPE. Equation (13) is used to compute IHC in tumor from the estimated IFVF ($\phi )$ and the prior knowledge of the tumor cell size (10 µm^29^). After reconstructing IFVF, EVF, and IHC maps, a 5 × 5 median filter was used to denoise the image.

### Computation of IFVF (${\varvec{\phi}})$ using scanning electron microscopy (SEM)

We used the method described by Bakay^12^ to compute the IFVF (referred as porosity for polyacrylamide phantoms) from SEM images. Binary gradient images of SEM images were computed using Canny edge detection algorithm and then dilation algorithm was applied using a disk-shaped structuring element. Porosity was calculated as the area covered by pores divided by total number of pixels.

### Estimation of IHC by ex vivo tumor infusion

A 3-dimensional ex vivo* infusion* technique similar to^6^ was used to provide independent confirmation of IHC. Tumors growing in mice were carefully excised and suspended completely immersed in an isotonic saline solution at room temperature. A needle was inserted into the center of the tumor and connected with transparent tubing to a reservoir of iso-osmotic albumin-containing fluid a fixed height (105 cm H_2_O) above the tumor. The steady-state infusion rate was calculated from the velocity of an air bubble introduced into the tubing at the start of the experiment. From Darcy's Law, interstitial conductivity was then estimated to be: *IHC* = *Q/*(*4πaP*), where *Q* is the steady-state infusion rate, *a* is the radius of the infusion cavity (assumed to be equal to the radius of the infusion needle) and *P* is the hydrostatic pressure.

### Theoretical analysis of the proposed methods

Equations (6) and (12) are used to compute $\phi$, and ${\phi }_{\mathrm{M}}$, respectively. Both equations do not require any initialization of $\phi$ and ${\phi }_{\mathrm{M}}$ which ensures the robustness of the method. To show the performance of the proposed models, we computed $\phi$ and ${\phi }_{\mathrm{M}}$ using our proposed models by taking Lame’s parameters ($\lambda$ and $\mu$) from^29^ and then compared with the extracellular volume fraction estimated by the finite element method. We also compared $\phi$ with the porosity value mentioned in^26^ for two different PR of the tumor. Percent Relative Error (PRE) is used to compare our results with the literature^26,29^.

### Performance metric

Quality of the estimated parameters was quantified using percent relative error (PRE). PRE is defined as^53^,

14 $$PRE = \sum\limits_{{c = 1}}^{C} {\sum\limits_{{r = 1}}^{R} {\frac{{abs\left( {\rho _{{v\left( {r,c} \right)}} - \rho _{e} \left( {r,c} \right)} \right)}}{{\rho _{{v\left( {r,c} \right)}} }} \times \frac{{100}}{{R \times C}}} }$$

where *ρ*_*v*_ is the validation parameter and *ρ*_*e*_ is the estimated parameter using our proposed models. *R* and *C* represent the row and column in the estimated maps, respectively. PRE analysis is performed only for the inclusion.

## Phantom experiments

Polyacrylamide phantoms can be used as good tissue mimic phantoms^54^ and were used to validate the proposed theories. Three non-uniform (with inclusion) phantoms were created using a combination of tofu and polyacrylamide gel following the protocol reported in^54^. In the non-uniform tissue mimicking phantoms, the background was made of tofu (Morinaga Nutritional Foods, Inc., Torrance, CA USA) while the inclusion (simulating the poroelastic tumor) was made of polyacrylamide gel. In three different phantoms the polyacrylamide inclusions were made with 1%, 4%, and 5% cross-linker, respectively, to simulate different fluid transport parameters. In all cases, the size of the phantom was 80 mm × 60 mm × 40 mm while the cylindrical inclusion diameter was 15 mm. Porosity of the polyacrylamide samples computed from SEM images were used to validate porosity estimated using our proposed methods. Intrinsic permeability (*k*) was also computed for the inclusion using the proposed method.

### Ultrasound poroelastography (USPE)

Elastography was carried out using a Sonix RP system (Ultrasonix, Richmond, BC, Canada) with a 38-mm linear array transducer, which operates with a center frequency of 6.6 MHz, bandwidth 5–14 MHz and beamwidth equal to 1 mm at the focus. Compression was applied from the top using different weights ranging 100–400 g. A compressor plate was attached to the transducer face to apply compression on a large area.

### Scanning electron microscopy (SEM)

For the SEM measurements, the polyacrylamide gels were freeze-dried using a lyophilizer (Freezone 4.5, Labconco, Kansas City, MO, USA) after freezing at − 80° overnight. Samples were mounted on an aluminum stub using conductive adhesive tape and sputter coated with a 10-nm layer of platinum using a high-resolution sputter coater (Cressington 208 HR, Cressington Scientific Instruments, UK). SEM was performed under high vacuum using a FEI Nova NanoSEM230 (FEI Company, Hillsboro, OR, USA) at a voltage of 10 kV and spot size 3.0 under a working distance of 5 mm^54^.

## In vivo experiments

**Figure 3:**
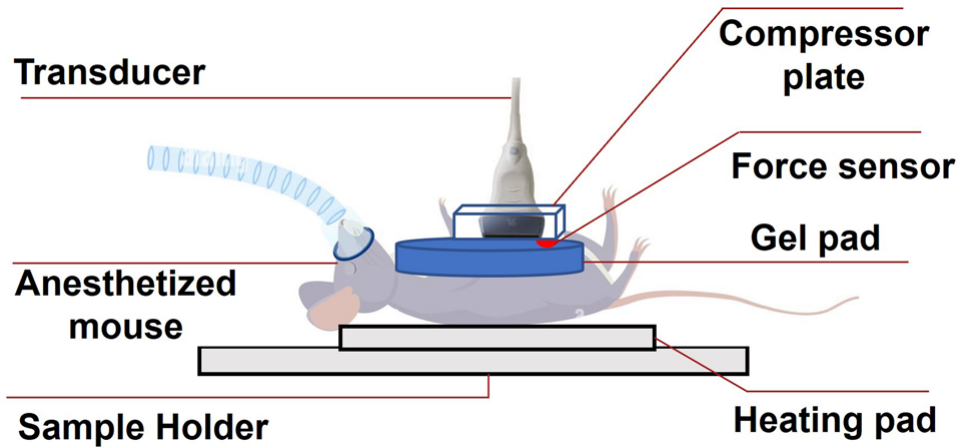
Schematic diagram of the in vivo experimental setup.

Twelve mice (6 untreated, 6 treated) implanted with triple negative breast cancer (TNBC) were scanned once a week for three subsequent weeks. MDA-MB-231 cell line obtained from Fisher Scientific (Waltham, MA, USA) was used in this study. The cancers were created by injecting cancer cells orthotopically in the mammary fat pad of immunocompromised female NOD/SCID gamma (NSG) mice^27^. In vivo experiments were approved by the Houston Methodist Research Institute, Institutional Animal Care and Use Committee (ACUC-approved protocol # AUP-0614-0033). All experiments were performed in accordance with relevant guidelines and regulations. Six mice were kept untreated, and the other six were treated with Epirubicin alone (n = 3), or LEPILOX (liposomes loaded with Epirubicin and conjugated with a targeting anti-LOX antibody on the particle surface, n = 3) for three weeks. The dose of each drug was 3 mg/kg body weight once a week. A schematic diagram of the in vivo experiment at setup is shown in Fig. 3.

Each ultrasound imaging session was 5 min long and several RF data acquisitions were obtained from the mice during this period. During the ultrasound imaging session, each mouse was anesthetized with isoflurane and kept lying on a thermostat-regulated heating pad sedated for the entire session. Elastography was carried out using a 38-mm linear array transducer (Sonix RP, Ultrasonix, Richmond, BC, Canada) with a center frequency of 6.6 MHz, 5–14 MHz bandwidth. To compensate for the surface geometry as well as improve the focus inside the superficial tumors, an aqueous ultrasound gel pad (Aquaflex, Parker Laboratories, NJ, USA) was placed between the compressor plate and the protuberance of the developed tumor. The sampling rate of data acquisition was configured as 0.1 s/sample A force sensor (Tekscan FlexiForce, manufactured by Tekscan, Inc., South Boston, MA, USA-02127) was inserted between the gel pad’s top surface and the compressor plate to record the applied force during the compression. A Microsoft Windows based interface software is provided with the sensor and can be used to observe and record the applied force. The sensor used in the kit is of model #A201, which senses a force range 0–4*.*4 N in a scale of 0–255. The diameter of the sensing area of the sensor is 9*.*53 mm. The area of the sensing area is calculated as 7*.*1331 × 10^−5^m^2^ (${A}_{r}=\pi {r}^{2}$)^45^. The applied stress in Pa is calculated by ${\sigma }_{0}=\frac{{F}_{r}\times 4.4}{255\times {A}_{r}},$ where, *F*_*r*_ is the force reading obtained from the sensor during the experiments. It is noted that *σ*_0_ is the axial component of the applied stress and the other two components (lateral and elevation) are zero. Creep compression was performed was manually on the animals, with the duration of each creep acquisition being one minute. Duration of the experiment selected based on the time constant of the soft tissue and tumor to ensure that at the end of the experiment, both the tumor and surrounding normal tissues become fully relaxed^55^. The ultrasound radio-frequency (RF) data acquisition was synchronized to the applied compression. Applied compression was monitored using a graphical user interface software purchased with the force sensor.

## Results

### Theoretical analysis results

Reconstructed $\phi$ and ${\phi }_{\mathrm{M}}$ from our proposed models using the Lame’s parameters ($\lambda$ and $\mu$) mentioned in^29^ are shown in Table 1. In^29^, $\lambda$ and $\mu$ are computed from ${\phi }_{\mathrm{M}}$ by finite element as well as analytical modeling. From Table 1, it can be seen that, computed ${\phi }_{\mathrm{M}}$ using our proposed method are very close to the reported values in^29^ (PRE less than 5%). To discuss the performance of the model in upper extremum of ${\phi }_{\mathrm{M}}$, $\nu$ is computed from $K$ and $\mu$ using ${\phi }_{\mathrm{M}}$ = 1 (meaning that the total tissue volume is occupied by the ECM). If ${\phi }_{\mathrm{M}}$ = 1, we found $\nu =$ 0.25, which is the PR of the ECM. However, $\nu$ decreases with increasing cell shear modulus and for rigid cells (shear modulus → ∞), $\nu$ is always less than 0.25, except when ${\phi }_{\mathrm{M}}$ = 0. If ${\phi }_{\mathrm{M}}$ = 0, total structure is occupied with rigid cells, $\nu$ is not defined. Reconstructed IFVF ($\phi$) is also compared with the data reported for tissue in literature^26^. At very high values (slightly less than 0.5) of $\nu$, $\phi$ is very low, shown in the table, in good correspondence with the values reported in Leiderman et al.^26^.

**Table 1:** Comparison of $\phi$, ${\phi }_{M}, k$ with literature value. $\lambda$ And $\mu$ are expressed relative to ${\mu }_{M}$.

$\lambda$ ^12^	$\mu$ ^12^	$K=\frac{3\lambda +2\mu }{3}$	$\nu =\frac{3K-2\mu }{6K+2\mu }$	${\phi }_{\mathrm{M}}$(EVF)			$\phi$(IFVF)		
				Equation (12)	^29^	PRE	Equation (6)	^26^	PRE
28.565	0.153	28.667	0.497	0.13	0.1	0.3	0.007	–	–
13.526	0.211	13.666	0.492	0.24	0.2	0.2	0.02	0.02	$\approx$ 0
8.484	0.274	8.666	0.484	0.34	0.3	0.13	0.04	–	–
5.937	0.345	6.167	0.472	0.46	0.4	0.15	0.07	0.06	0.16
4.385	0.423	4.667	0.456	0.56	0.5	0.12	0.12	–	–
3.326	0.511	3.666	0.433	0.65	0.6	0.08	0.17	–	–
2.546	0.61	2.952	0.403	0.74	0.7	0.05	0.25	–	–
1.935	0.722	2.416	0.364	0.83	0.8	0.04	0.34	–	–
1.433	0.851	2.000	0.313	0.91	0.9	0.01	0.45	–	–

### Phantom experimental results

**Figure 4:**
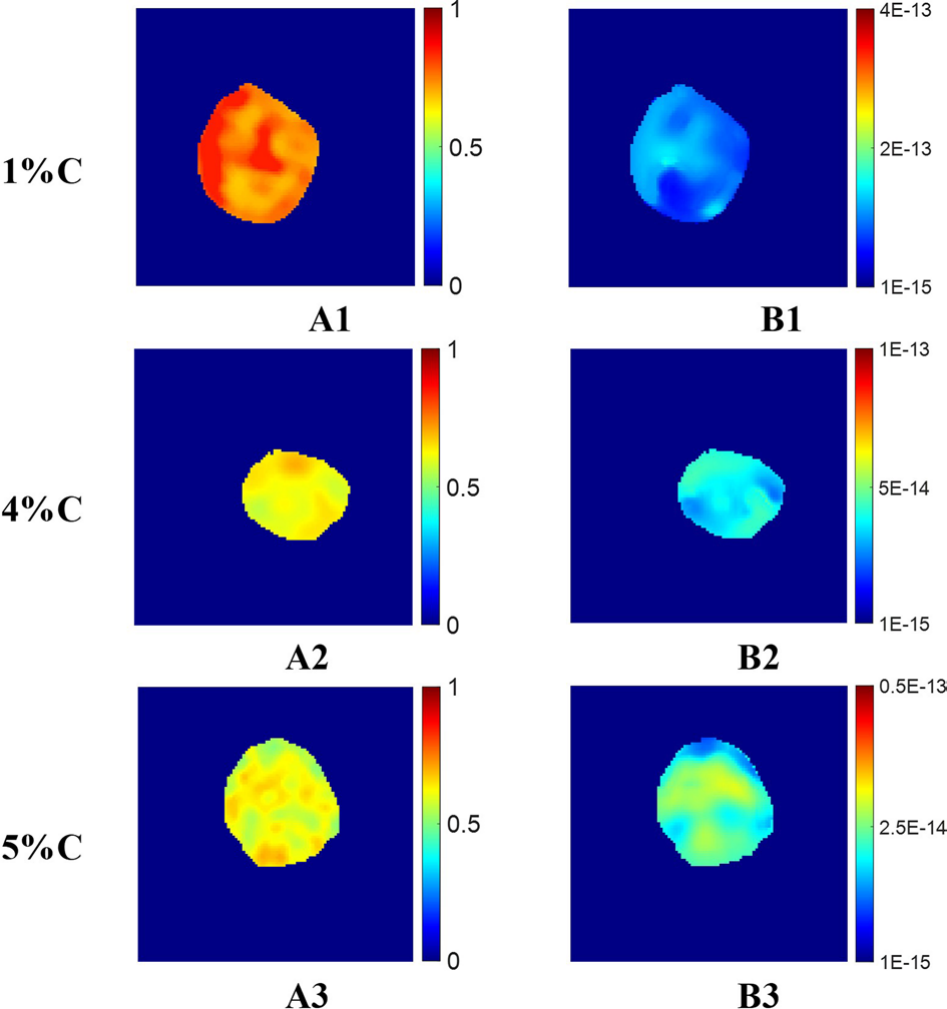
(**A1**–**A3**) Estimated IFVF using the proposed method for 1%, 4%, and 5%C polyacrylamide phantoms, (**B1**–**B3**) estimated corresponding intrinsic permeability (*k*) maps. Unit of *k* is m^2^.

**Figure 5:**
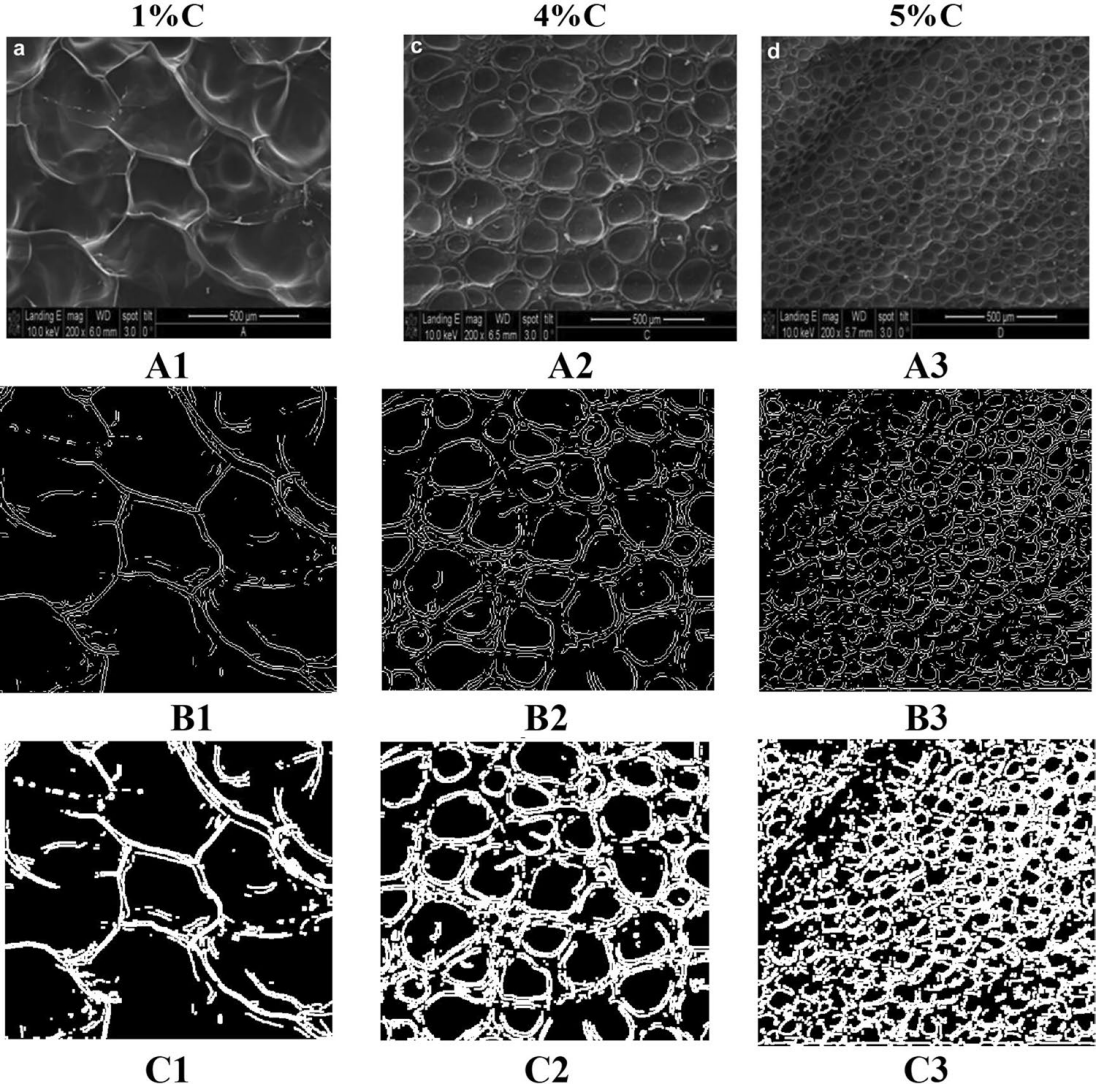
(**A1**–**A3**) SEM images, (**B1**–**B3**) Binary gradient images (**C1**–**C3**) dilated gradient images.

Axial and lateral strains were computed from RF data for the nonuniform polyacrylamide samples. YM, PR were computed from the axial strain, lateral strain, and applied stress using Eshelby’s method^39^. Porosity (IFVF) and intrinsic permeability (*k*) were computed using the proposed approach using Eqs. (6), and (12), respectively. Assuming polyacrylamide gels as random beds with pore volume less than 0.9, ${c}_{kc}$ is considered 5^40^ to compute *k.* Reconstructed IFVF and intrinsic permeability images were denoised using a 5 × 5 median filter. Images of the estimated parameters of three phantoms are shown in Fig. 4. SEM images along with the binary gradient and dilated binary gradient images for the same phantoms are shown in Fig. 5A1–A3,B1–B3,C1–C3, respectively. Mean values with one standard deviation (STD) of estimated mean porosity inside the inclusion for 1%, 4%, and 5% cross-linker percentages using poroelastography and SEM, and estimated Intrinsic permeability (*k*) using poroelastography are shown in Table 2. We found no significant difference between the porosity measurements obtained using ultrasound and the corresponding ones obtained using SEM (PRE less than 10%). An empirical model to compute intrinsic permeability from the cross linker percentage of polyachrylamide was developed in^56^ as, $k=0.0294 {C}^{-1.850}$, where $C$ is the percentage of crosslinker. Computed k for polyacrylamide phantoms of three cross-linkers using our proposed method and using empirical model^56^ are shown in Table 2 for comparison. We observe our permeability estimates match well the literature data (same order of magnitude and the same trend for three % of cross-linkers.

**Table 2:** Estimated porosity and intrinsic permeability (*k*) for different %C of polyacrylamide phantoms (inclusion).

% Cross linker	Porosity			*k* (10^–14^ m^2^)	
	USPE Mean ± SD	SEM	PRE	USPE Mean ± SD	^56^
1%	0.760 ± 0.050	0.858	8.04	8.15 ± 2.75	$\approx$ 2.94
4%	0.622 ± 0.026	0.677	7.06	3.14 ± 0.46	$\approx$ 1.13
5%	0.609 ± 0.037	0.609	0.04	2.30 ± 0.45	$\approx$ 0.75

### In vivo experimental results

**Figure 6:**
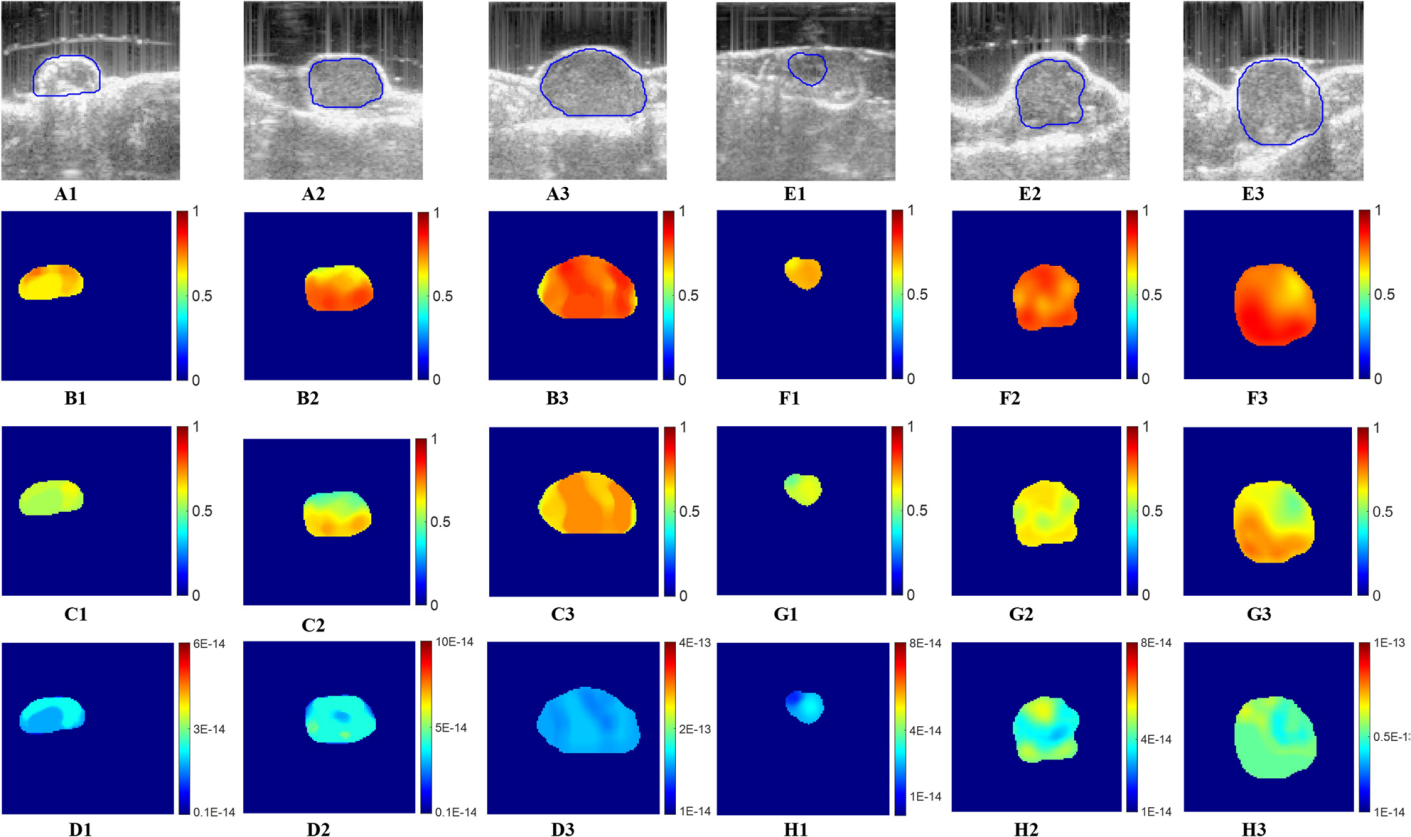
Estimated IFVF, EVF and IHC maps for two untreated mice using USPE in week1, week2, and week3. (**A1**–**A3**) BMode images of mice #1 at three time points, respectively. (**B1**–**B3**) EVF images, (**C1**–**C3**) IFVF images, and (**D1**–**D3**) IHC images of mice #1 at three time points. Similarly, (**E1**–**E3**) BMode images, (**F1**–**F3**) EVF, (**G1**–**G3**) IFVF, and (**H1**–**F3**) IHC images for mice #2 at three time points. Unit of IHC is m^2^ (Pa.s)^−1^. EVF and IFVF are dimensionless.

**Figure 7:**
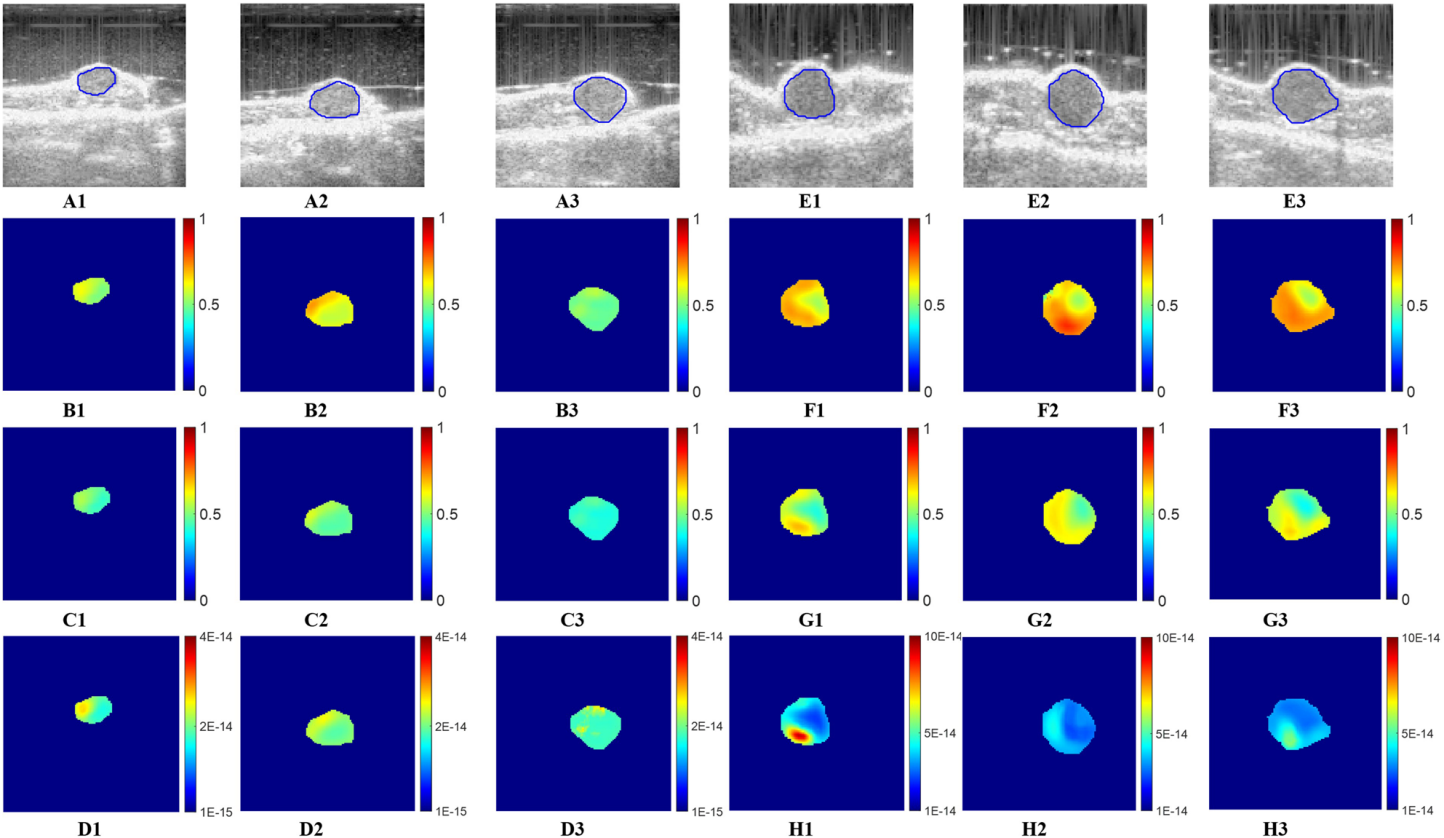
Estimated IFVF, EVF and IHC maps for two treated mice using USPE in week1, week2, and week3. (**A**1–**A3**) BMode images of treated mice #1 at three time points, respectively. (**B1**–**B3**) EVF images, (**C1**–**C3**) IFVF images, and (**D1**–**D3**) IHC images of mice #1 at three time points. Similarly, (**E1**–**E3**) BMode, (**F1**–**F3**) EVF, (**G1**–**G3**) IFVF, and (**H1**–**F3**) IHC images for mice #2 at three time points. Unit of IHC is m^2^ (Pa.s)^−1^. EVF and IFVF are dimensionless.

In vivo study reported in this paper is in accordance with ARRIVE guidelines (Animal Research: Reporting of In Vivo Experiments). Reconstructed IFVF, EVF, and IHC images along with the B-mode images for two untreated tumor cases (mice #1, and mice #2) at three time points (week 1, week 2 and week 3) are shown in Fig. 6. Figure 6(A–D) presents BMode images (A1–A3), estimated EVF maps (B1–B3), IFVF maps (C1–C3), and IHC (D1–D3) maps for mice #1 at three weeks (1, 2, 3). Figure 6(E–H) presents BMode images, EVF maps, IFVF maps, and IHC maps for mice #2, respectively. Week1, week2, and week3 data are represented by the columns in Fig. 6(1, 2, 3). Similarly, BMode images, and three estimated parameters (EVF, IFVF, and IHC) for two treated tumors (mice #1: A, B, C, D and mice #2: E, F, G, H) at three time points (1, 2, 3) are shown in Fig. 7.

Mean value of the estimated parameters (EVF, IFVF, and IHC) inside the tumors (two untreated and two treated) with SD about the mean are reported in Table 3. We see from the reconstructed IFVF results that for the untreated cases, IFVF increases with time as the cancer grows. Such increment of IFVF correlates with observations from the literature^18,33^. Similar observations hold for the other untreated cases. In contrast to the untreated cases, IFVF does not change with time significantly. Such reduction of IFVF may be a result of the applied treatment and it may be related to the reduction of IHC^33^. Estimated IFVF correlates with the literature reported in^3^ for different types of tumor tissues. Similar to the IFVF, the EVF increases with time for untreated tumors whereas EVF decreases or increases in a very insignificant manner in treated tumors. EVF value is slightly more than the IFVF value because EVF includes the interstitial space with collagen fibers, elastin fibers etc. whereas IFVF includes only the interstitial fluid. In case of estimated IHC, it increases significantly for the untreated tumors with time. For treated tumors on the other hand, IHC does not increase significantly. In our orthotopic mouse model, estimated IHC for the untreated tumor is in the range of 0.11 × 10^–13^ to 0.85 × 10^–13^ m^2^ (Pa.s)^−1^, in between the range reported in literature^6–8,13^.

**Table 3:** Mean ± SD of estimated extracellular volume fraction (EVF), interstitial fluid volume fraction (IFVF), and interstitial hydraulic (IHC) conductivity inside tumors for two untreated and two treated in vivo tumors in three time points. HC is presented in (m^2^ (Pa.s)^−1^).

Time points	Untreated						Treated					
	(M #1)			(M #2)			(M #1)			(M #2)		
	EVF Mean ± SD	IFVF Mean ± SD	IHC*,* 10^–14^ m^2^ (Pa.s)^−1^ Mean ± SD	EVF Mean ± SD	IFVF Mean ± SD	IHC, 10^–14^ m^2^ (Pa.s)^−1^ Mean ± SD	EVF Mean ± SD	IFVF Mean ± SD	IHC*,* 10^–14^ m^2^ (Pa.s)^−1^ Mean ± SD	EVF Mean ± SD	IFVF Mean ± SD	IHC, 10^–14^ m^2^ (Pa.s)^−1^ Mean ± SD
Week1	0.61 ± 0.08	0.55 ± 0.02	1.99 ± 0.30	0.62 ± 0.02	0.56 ± 0.03	2.43 ± 0.48	0.55 ± 0.03	0.45 ± 0.04	2.04 ± 0.32	0.62 ± 0.05	0.54 ± 0.07	4.45 ± 0.15
Week2	0.72 ± 0.06	0.59 ± 0.07	3.86 ± 0.47	0.70 ± 0.03	0.61 ± 0.03	4.17 ± 0.51	0.59 ± 0.06	0.47 ± 0.03	2.32 ± 0.16	0.64 ± 0.07	0.57 ± 0.05	3.54 ± 0.47
Week3	0.75 ± 0.04	0.64 ± 0.03	14.22 ± 0.70	0.73 ± 0.06	0.64 ± 0.07	8.33 ± 1.72	0.49 ± 0.02	0.42 ± 0.02	1.87 ± 0.16	0.65 ± 0.05	0.54 ± 0.07	3.74 ± 0.60

**Figure 8:**
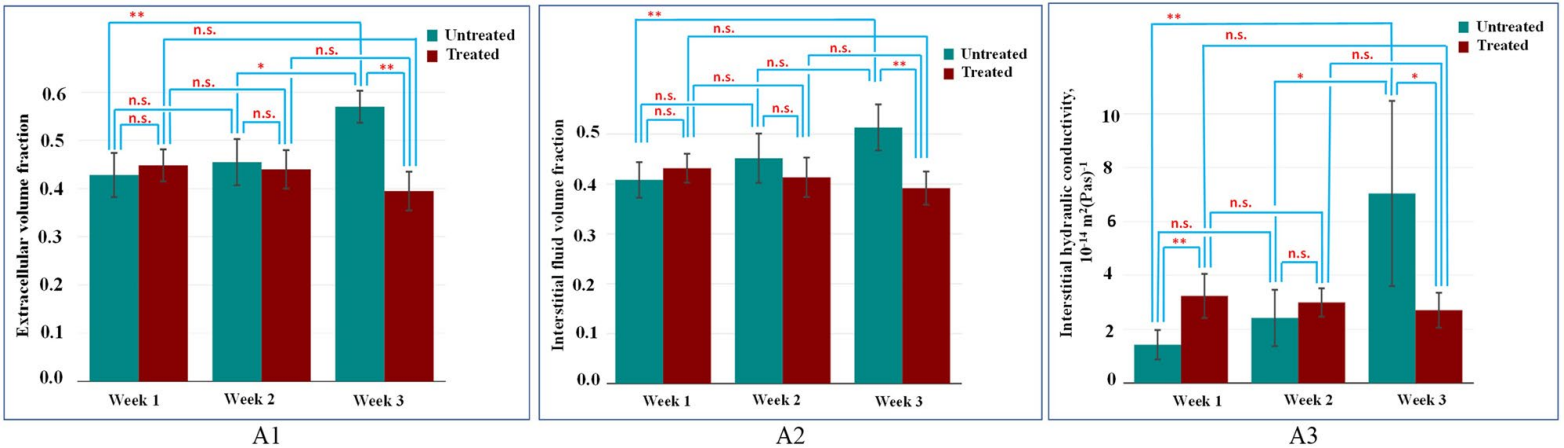
(**A1**) Mean EVF values for the treated and untreated in vivo tumors at week 1, week 2 and week 3. (**A2**) Mean values of IFVF for the treated and untreated in vivo tumors at week 1, week 2 and week 3. (**A3**) Mean values of IHC for the treated and untreated in vivo tumors at week 1, week 2 and week 3. n.s. means not statistically significant. One, two and three stars correspond to p value less than 0.05; 0.01; 0.001, respectively.

The mean values with the corresponding standard deviations of EVF, IFVF, and IHC for six treated mice and six untreated mice used in our in vivo experiments at the three different time points (week 1, week 2 and week 3) are shown as bar graphs in Fig. 8(A1–A3, respectively). In the first week, the mean EVF, IVF, and IHC of the tumors in the untreated mice were found to be below 0.45, 0.41, and 2 × 10^–14^ m^2^ (Pa.s)^−1^. In the second week, the mean values of all these three parameters of the untreated tumors increased slightly (above 0.46, above 0.42, and above 2.4 × 10^–14^ m^2^ (Pa.s)^−1^, respectively). In the third week, these parameters of untreated mice were found to be increased very significantly (above 0.55, above 0.5, and above 7 × 10^–14^ m^2^ (Pa.s)^−1^, respectively). On the other hand, the mean EVF, IFVF, and IHC of the treated tumors were found to be in slight decreasing order or not significantly different over the three weeks.

### Ex vivo experimental results

**Figure 9:**
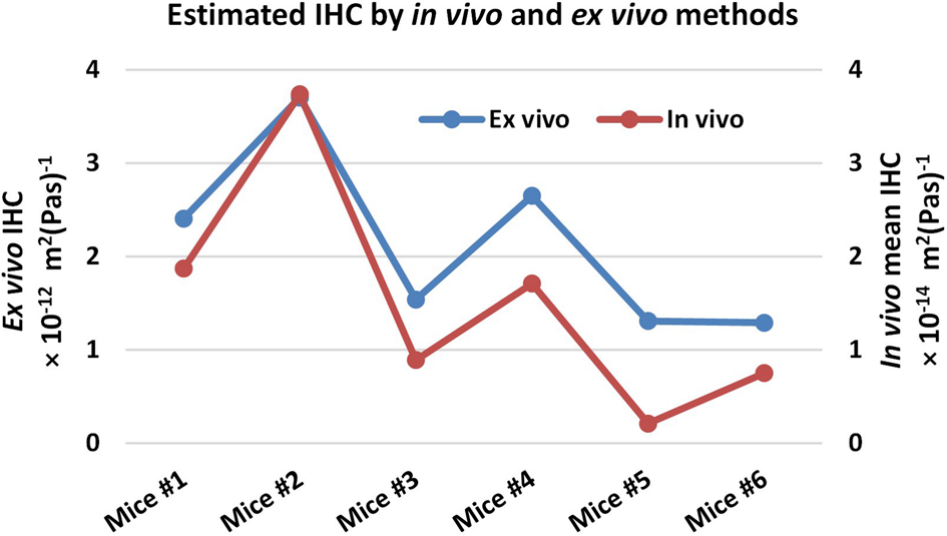
Estimated mean IHC using in vivo and ex vivo techniques.

Ex vivo experiment was performed on the six treated mice euthanizing with isoflurane overdose after completing the last imaging session in week 3. Our measured IHC range for six ex vivo tumors was found (1.24–5.81) × 10^–12^ m^2^ (Pa.s)^−1^, as shown in Table 4, is in the range of reported IHC in ex vivo^8^. In^8^, IHC was measured from excised tumor in human colon adenocarcinoma, human glioblastoma, human soft tissue sarcoma, and murine mammary carcinoma grown in mice and reported as: (1.86 ± 0.57) × 10^–12^ for carcinoma, (3.37 ± 2.06) × 10^–12^ for adenocarcinoma, (4.87 ± 2.25) × 10^–13^ for glioblastoma, and (0.69 ± 0.53) × 10^–13^ m^2^ (Pa.s)^−1^ for sarcoma. Ex vivo experiments were done in this study to compute IHC using the fluid infusion technique^6^ and corroborate the in vivo IHC measurements. Based on the experimental results obtained on five ex vivo tumors, the mean ex vivo IHC was found higher than the mean in vivo IHC (Fig. 9). Ex vivo results provide a single estimate of IHC while multiple measurements would provide an overall better assessment of this parameter. Nonetheless, a reasonable agreement between the in vivo and ex vivo results has been observed in this study (a linear correlation with *R*^*2*^ > 0.94).

**Table 4:** Estimated interstitial hydraulic conductivity values by ex vivo infusion experiments for treated tumors.

Mice #	1	2	3	4	5	6
IHC, m^2^ (Pa.s)^−1^	2.408 × 10^–12^	3.705 × 10^–12^	1.54 × 10^–12^	2.65 × 10^–12^	1.309 × 10^–12^	1.29 × 10^–12^

## Discussion

In this paper, novel non-invasive ultrasound poroelastography methods to image the interstitial fluid volume fraction (IFVF), extracellular volume fraction (EVF), and interstitial hydraulic conductivity (IHC) in cancers in vivo have been proposed. These properties are important indicators of malignancy and tumor aggressiveness, but they are difficult to measure in vivo, especially non-invasively. Current methods to measure hydraulic permeability, such as wick-in needle, are invasive^1,6,8^ and do not provide localized measures of this parameter. Hydraulic conductivity can be measured via intravital imaging in tumors using optical imaging^57^, but optical methods have limited penetration. Magnetic resonance imaging techniques can be used to assess some of the poroelastic parameters^15–17^, but these methods are costly, computationally expensive and require use of contrast agents. The proposed ultrasound-based method to estimate these parameters is non-invasive, fast, cost-effective and provide localized tissue measurements. These methods may be easily translatable into the clinics, given the wide availability of diagnostic ultrasound systems.

Selected proposed methods were validated using SEM in controlled polyacrylamide phantoms while in vivo feasibility was tested in a tumor animal model. Overall, our preliminary in vivo results show that all parameters increased significantly from week 1 to week 3 in the untreated group while they show a decreasing trend (albeit not statistically significant) in the treated group. These findings would suggest that the administered treatment affected the EVF/IFVF/IHC in the tumors. This observation correlates with prior studies reported in the literature, according to which, administration of drugs can cause a reduction of interstitial fluid transport^3^. In vivo experiments reported in this paper resulted in EVF of 40–55% (see Fig. 8-A2), which is in between the range 36 and 60% for tumors implanted in mice reported in the literature^3^. Similarly, in vivo IHC values in this proposed study were found in the range of (0.22–0.64) × 10^–14^ m^2^ (Pa.s)^−1^, which is in between the IHC values reported for tumors in previous studies (4.20 × 10–15 m^2^ (Pa.s)^−1^ to 5.92 × 10^–13^ m^2^ (Pa.s)^−1^^26^).

The main limitations of the proposed method reside in the assumptions inherent to the models developed to quantify the parameters. To assess IFVF, a bi-phasic poroelastic model with solid phase fully saturated by the interstitial fluid (IF) is assumed. All other constituents of the tissue other than the IF are assumed as the solid phase on our considered length scale. Both solid and fluid phases are assumed to be incompressible. These assumptions are consistent to prior studies pertaining to cancer mechanics, medical imaging and drug delivery^1,25,26^. However, this assumption may be valid in the sense that IF primarily consists of water. Solid phase incompressibility may come from the fact that tissue deformation is mainly caused by the fluid movement rather than the movement of the solid part.

To model EVF, tumor tissue is assumed as an array of incompressible cells, surrounded by compressible ECM consisting of an elastic isotropic mesh of randomly oriented interconnected fibers. This assumption was also made in^29^ for soft tissue modeling. The cell membranes are relatively impermeable to water. For mammalian cell membranes (other than erythrocytes), the hydraulic permeability is typically of the order 10*–*13 m/s/Pa^5^. Therefore, the cells are assumed to deform in an incompressible manner. On the other hand, cells in very soft tissues deform readily in response to shear stress. The contribution of cells to the total stress occurring in the connective tissues is generally small^29^. Shear modulus of ECM is much higher than the shear modulus of cell in soft tissue^29^. In real tissues, cell arrangements are random to some extent, and regular cubic cells are unlikely to occur^58^. In^29^, it was shown that the tissue elastic parameters are not very sensitive to cell shape. Therefore, the assumption of regular cell shapes may not be a major source of error. In the future, the proposed approach could be extended to compressible cells surrounded by anisotropic extracellular matrix^58^.

A limitation to model IHC inside cancers may be due to the assumption of the shape and size of tumor cell to compute specific surface area (*S*) of the cells. Assuming ellipsoidal shape for cell, *S* is computed using the average diameter of the cell. We use average cell diameter to be 10 µm, based on^29^. Another limitation is the assumption of Kozeny–Carman constant ($${c}_{kc})$$, required to compute IHC.

We note that our methods to estimate the EVF and IFVF require the knowledge of YM and PR in the tumors. To estimate YM and PR, we used Eshelby’s theory for elliptical tumors^46^. YM and PR are important indicators for tumor diagnosis and prognosis^52,59^. Likewise, poroelastic material properties such as interstitial hydraulic conductivity (IHC), vascular permeability (VP), extracellular volume fraction (EVF), interstitial space fraction (IVF), etc. are important markers of tumor progression and may be used to assess the efficacy of treatments^5,25,52,60,61^. These poroelastic properties can have substantial impact on drug delivery to the tumor. For example, an increase in IHC creates an increase of IFP, which is an important physiological barrier for drug delivery. Moreover, large interstitial space is generally correlated to the leakiness of lymphatic networks in tumor, which are also barriers for drug molecules due to the relatively long distance a drug molecule needs to cross before reaching the tumor cell.

Elevated interstitial fluid pressure (IFP) and solid stress (SS) are physical hallmarks of cancer^62^ and co-evolve during tumor growth^63^. This co-evolution happens within relatively long-time intervals, which are orders of magnitude longer than an elastographic experiment’s time interval (~ 1 min). Solid stresses, which can be compressive or tensile^64^, can impact the pore size over time. Pore size is related to the pore density ($\epsilon )$ and porosity ($\phi$, defined as IFVF in this study) by $\epsilon =\frac{2N}{\uppi {V}_{b}}(\frac{{A}^{2}}{P})$ and $\phi =\frac{4\pi }{3}\epsilon$, where, $N/{V}_{b}$ is the number of pores in the bulk sample volume ${V}_{b}$. Alteration in pore size is unlikely to change the pore density in bulk sample (our and most of the poroelastic models retrievable in the literature assume the tissue as a matrix with fully saturated pores so that continuum mechanics can be used)^3^. However, the elevated IFP and SS could affect the estimation of bulk modulus and shear modulus of the medium, and, therefore, may impact the estimated poroelastic parameters in this paper (IFVF, EVF, and IHC). Bulk modulus and shear modulus have been computed in this study using the Eshelby’s inclusion theory proposed in^45^ assuming that no elevated IFP and SS are present inside the tumor. Elevated IFP and SS inside the tumor could alter the strains experienced by the tumor and, therefore, would affect the estimation of the poroelastic measurements in this study. It is reasonable to expect that elevated IFP and SS may not affect the estimates of the poroelastic parameters as long as they are much smaller than the applied stress in the poroelastography experiment (1–5 kPa^46,52^). However, in cases where IFP and SS are significantly high and/or comparable to the applied poroelastography stress, a correction in the computation of the bulk and shear moduli may be required to obtain accurate estimates of the poroelastic parameters. This can be investigated in future studies.

Another challenge when computing poroelastic properties in vivo may be heterogeneities in solid stress and stiffness inside the tumor. These heterogeneities may result in heterogeneities in the estimated elastic moduli of the tumor. We consider the ensemble of interstitial, vascular and cellular spaces as a continuous, deformable solid phase saturated with a fluid phase consisting of water and macromolecular constituents. We define as “elementary volume” a volume of tissue large enough to contain a sufficiently large number of cells and blood vessels. The dimension of the elementary volume is at least one order of magnitude larger than the distance between the vessels (< 100 µm)^65^. As the proposed method to estimate poroelastic properties is capable of estimating EVF, IFVF, IHC on a local basis, the estimates should be reflective of underlying heterogeneous mechanical properties.

## Conclusion

In this paper, novel non-invasive ultrasound poroelastography techniques for imaging the extracellular volume fraction (EVF), interstitial fluid volume fraction (IFVF), and interstitial hydraulic conductivity (IHC) in cancers in vivo are proposed and analyzed. EVF, IFVF, and IHC are clinically significant parameters, which carry important information for cancer diagnosis and drug delivery. In preliminary in vivo experiments, estimated EVF, IFVF, and IHC were found to be significantly higher in untreated tumors than treated tumors. Based on the importance of these parameters in cancer treatment and widespread availability of ultrasound imaging systems, the developed methods may become a useful alternative option to current methods.

## Data Availability

The datasets generated during and/or analyzed during the current study are available from the corresponding author on reasonable request.
